# AMR battle in Pakistan: from national action plans to local failures

**DOI:** 10.1186/s13690-025-01568-6

**Published:** 2025-04-08

**Authors:** Zikria Saleem

**Affiliations:** Department of Pharmacy Practice, Faculty of Pharmacy, Bahauddin Zakariya University, Multan, 60800 Pakistan

**Keywords:** AMR, Pakistan, National action plans

## Abstract

This correspondence highlights critical gaps in Pakistan’s response to antimicrobial resistance (AMR) five years after implementing the National Action Plan. Despite extensive policy discussions, workshops, and official reports, meaningful actions remain insufficient. Key issues include a lack of awareness among healthcare professionals about resistance patterns, inadequate surveillance, poor infection prevention and control practices, and a pharmaceutical industry focused more on producing branded generics than innovating new antibiotics. The correspondence calls for urgent action to bridge the gap between policy and practice, emphasizing the need for comprehensive awareness, better surveillance, and stronger regulatory enforcement to contain AMR effectively in Pakistan.

**Table:** 

**Text box 1. Contributions to the literature**
• Persistent gaps in Pakistan’s AMR response, emphasizing the disconnect between policy frameworks and on-the-ground realities.
• Provides critical insights into the impact of inadequate surveillance and irrational antibiotic use, reinforcing the need for localized prescribing guidelines.
• Contextualizes Pakistan’s AMR challenges within the broader global AMR landscape, demonstrating the need for coordinated, multisectoral action.
• Calls for pragmatic solutions and governmental accountability, advancing discussions on actionable strategies to strengthen AMR management in resource-limited settings.

Antimicrobial resistance (AMR) poses one of the most severe public health threats globally, with significant implications for health systems, economies, and societies. In response to the global action plan on AMR, Pakistan developed its National Action Plan (NAP) in 2017, with the National Institute of Health (NIH) serving as its custodian. Five years on, a review of the progress reveals a concerning gap between the rhetoric of policy documents, official reports, workshops and conferences, and the realities on the ground, where meaningful actions remain woefully inadequate [1]. A critical issue is the lack of awareness about AMR, not only among the general public and but alarmingly among healthcare professionals themselves. Physicians, who are the frontline defense against AMR, often lack knowledge of the resistance patterns of common pathogens, leaving them to make treatment decisions based on outdated or generalized information. Many rely on personal experience or the influence of pharmaceutical companies rather than evidence-based practices, leading to poor antibiotic stewardship [2]. Therefore, effective surveillance is a cornerstone of any strategy to mitigate AMR, yet Pakistan’s efforts in this area are dismally inadequate. For instance, the exact resistance patterns and causative agents for common infections, such as community-acquired pneumonia, remain poorly understood, which is essential for guiding treatment protocols and informing public health policies. This lack of data hampers effective treatment and fuels the inappropriate empirical use of broad-spectrum antibiotics, further exacerbating resistance [3]. The failure to enforce rational antibiotic use is further compounded by the lack of locally adapted prescribing guidelines. Public behaviours, such as seeking treatment from unqualified practitioners and self-medicating with leftover antibiotics, driven by financial constraints and inadequate public education, perpetuate irrational antibiotic use in Pakistan [4]. This situation is exacerbated by the poor legislation and weak regulatory enforcement, where the dispensing of antibiotics without proper prescription is rampant [5]. Another glaring issue is the poor state of infection prevention and control (IPC) practices in Pakistan. Surgical prophylaxis, for example, is a significant area of concern. While international guidelines recommend a single preoperative dose of first generation cephalosporins, in Pakistan, it is not uncommon for patients to receive broad-spectrum antibiotics for two to three days before surgery, continuing for up to five days postoperatively [1]. The disparity between private and government healthcare practices exacerbates AMR. Moreover, a concerning trend in Pakistan’s pharmaceutical industry is its heavy focus on producing branded generics of existing molecules with serious quality concerns as poorly regulated by drug regulatory authority of Pakistan (DRAP). This has led to an oversaturation of the market with broad spectrum antibiotics like 3rd generation cephalosporins and quinolones, with hundreds of brands available [6]. It is crucial to establish mechanisms within DRAP to regularly revise marketing authorizations. For antibiotics, restrictions should align with AMR priorities, such as strict controls on Reserve antibiotics, moderate restrictions on Watch antibiotics, and promoting the rational use of Access antibiotics to minimize resistance development.

The situation in Pakistan underscores the urgent need to move beyond discussions and policy documents to tangible action. The NAP on AMR, while a step in the right direction, has yet to translate into meaningful progress on the ground. Addressing AMR in Pakistan requires robust surveillance to understand resistance patterns, inform guideline updates, enforce IPC measures and improve prescriptions at all healthcare levels. Tackling over-the-counter antibiotic dispensing and launching behavioural science-based awareness campaigns for both the public and healthcare professionals are also essential steps. Examples of successful AMR interventions from African countries, such as stewardship programs, stricter regulatory frameworks, and awareness campaigns, provide practical insights and a roadmap for addressing similar challenges in Pakistan [7]. Addressing AMR in Pakistan requires a multifaceted approach that goes beyond rhetoric. It is time for the government, healthcare providers, and the pharmaceutical industry to take responsibility and act decisively. Failure to do so will result in a public health catastrophe of unprecedented scale, not just for Pakistan but for the global community.

## Data Availability

No datasets were generated or analysed during the current study.

## Declarations

### Disclaimer

Used AI tools to improve language.
